# Required concentration index quantifies effective drug combinations against hepatitis C virus infection

**DOI:** 10.1186/s12976-020-00135-6

**Published:** 2021-01-09

**Authors:** Yusuke Kakizoe, Yoshiki Koizumi, Yukino Ikoma, Hirofumi Ohashi, Takaji Wakita, Shingo Iwami, Koichi Watashi

**Affiliations:** 1 Department of Biology, Faculty of Sciences, Kyushu University, Fukuoka, 812-8581 Japan; 2 Present address: Data Science Group, Advanced Technology Division, INTAGE Inc, Tokyo, 101-8201 Japan; 3 National Center for Global Health and Medicine, Tokyo, 162-8655 Japan; 4 Department of Virology II, National Institute of Infectious Diseases, Tokyo, 162-8640 Japan; 5 Department of Applied Biological Science, Tokyo University of Science, Noda, 278-8510 Japan; 6 Institute for the Advanced Study of Human Biology (ASHBi), Kyoto University, Kyoto, 606-8501 Japan; 7 NEXT-Ganken Program, Japanese Foundation for Cancer Research (JFCR), Tokyo, 135-8550 Japan; 8 Science Groove Inc, Fukuoka, Japan; 9 Institute for Frontier Life and Medical Sciences, Kyoto University, Kyoto, Japan

**Keywords:** Mathematical model, Dose-response curve, Drug combination, Hepatitis C virus (HCV)

## Abstract

Successful clinical drug development requires rational design of combination treatments based on preclinical data. Anti-hepatitis C virus (HCV) drugs exhibit significant diversity in antiviral effect. Dose-response assessments can be used to determine parameters profiling the diverse antiviral effect during combination treatment. In the current study, a combined experimental and mathematical approaches were used to compare and score different combinations of anti-HCV treatments. A “required concentration index” was generated and used to rank the antiviral profile of possible double- and triple-drug combinations against HCV genotype 1b and 2a. Rankings varied based on target HCV genotype. Interestingly, multidrug (double and triple) treatment not only augmented antiviral activity, but also reduced genotype-specific efficacy, suggesting another advantage of multidrug treatment. The current study provides a quantitative method for profiling drug combinations against viral genotypes, to better inform clinical drug development.

## Introduction

Newly approved antiviral drugs rely upon dosage, treatment period, and drug combinations established during clinical trials. Trials require large cohorts of patients, significant cost, extensive time and strict management of ethics and compliance: Different dose regimens, treatment times and drug combinations are evaluated during trials [1, 2]. Additional trials are needed to establish drug efficacy against different viral genotypes [3–6]. Despite the significant effort placed in clinical trials, escalation of dosage, increased treatment period, and combination therapy, significant improvement in efficacy have not always been realized.

Drug concentrations which achieve 50% virus reduction (*IC*_50_), can be used to characterize drug activity. Lower *IC*_50_ means that antiviral effects are achieved with lower concentrations of drug [7]; however, a lower *IC*_50_ does not necessarily translate to higher antiviral effect. Antiviral effect depends on the Hill coefficient (*m*), in addition to *IC*_50_. A higher *m* value exponentially increases antiviral activity at higher doses [8–14]. We have previously shown that *m* is unique to each anti-hepatitis C virus (HCV) drug, and that augmentation of antiviral activity with escalation of drug dose is quite diverse among the types of anti-HCV drugs [14]. Multidrug treatments also result in diverse effects depending on the drug combination. In-depth profiling of drug antiviral effects can be useful in designing a treatment protocol with maximal antiviral efficacy. Such profiling could result in significant savings in clinical trials. To date, antiviral efficacy variances between different anti-HCV drugs and drug combinations has not been characterized in detail.

HCV infection is a leading cause of liver cirrhosis and hepatocellular carcinoma, serious public health problems affecting approximately 170 million people worldwide [15]. Recently, the development of new antiviral drugs known as direct acting antivirals (DAAs), have greatly improved treatment outcomes [16, 17]. Commercial interests restrict the combinations which have entered clinical trials as the combinations are all company specific rather than based on any assessment of what would be the best combination for all available agents. Further evaluation of HCV DAA effects could help identify the “best” available therapy and assist with optimizing combination treatments. A new quantitative method could also support evaluation of next generation anti-HCV treatments that could lead to the eradication of HCV. In the current study, we compare antiviral profiles of different classes of anti-HCV drugs to understand diversity of effects.

We recently developed a cell culture system combined with a mathematical model for quantifying anti-HCV drug efficacy at any concentration and multidrug combination [14]. We systematically evaluated and compared the intrinsic anti-HCV activity of 15 antiviral agents and their combinations against HCV genotype 1b. In the current study, we evaluate intrinsic anti-HCV activity in both genotype 1b and 2a. We create an “effectiveness” ranking for HCV replication inhibition in mono- and multi-drug cultures following exposure to high drug dose ranges. Significant diversity was observed between the antiviral activity profiles of different drugs. Thus, it is necessary to carefully select multidrug combinations to increase drug efficacy. We have demonstrated that the developed ranking index is able to delineate the advantages of past first-in-line anti-HCV treatment choices [14]. Thus, in the current study, we use the combined cell culture plus mathematical modeling approach to quantify efficacy of diverse antiviral drug combinations. This framework could be applied to other diseases requiring multidrug treatment, such as tuberculosis and cancer.

## Methods

Anti-HCV effect of each drug against genotype 1b or genotype 2a was evaluated with subgenomic replicon systems. As a genotype 1b model, LucNeo#2 (LN2) cells were employed that carry a dicistronic subgenomic replicon including open reading frames (ORFs) for the firefly luciferase-neomycin phosphotransferase fusion protein (translated by HCV 5′-untranslated region) and the NS3–NS5B region of HCV genotype 1b strain NN (translated by encephalomyocarditis virus (EMCV) internal ribosome entry site) [18]. Huh-7.5.1 cells transfected with a subgenomic replicon that included the ORFs for the NS3–NS5B region of HCV genotype 2a strain JFH-1 and the firefly luciferase gene (SGR-JFH1/Luc) were used for a genotype 2a model [19]. These cells were seeded at 7 × 10^3^ cells per well and treated with indicated concentrations of various drugs. Following 72 h of incubation, cells were lysed and cellular luciferase activity was measured to evaluate the HCV replication activity with a Luciferase Assay System (Promega) per manufacturer’s protocol [18].

Fourteen anti-HCV drugs were evaluated as single treatments. Eleven of these were direct-acting antivirals (DAAs) of the following classes: NS3/4A protease inhibitors [PIs: telaprevir (TPV), danoprevir (DPV), simeprevir (SMV), and asunaprevir (ASV)], nucleoside NS5B polymerase inhibitor [NI: sofosbuvir (SOF)], non-nucleoside NS5B polymerase inhibitors [NNIs: VX-222 (VX), dasabuvir (DAS), nesbuvir (NSV), and tegobuvir (TGV)], and NS5A inhibitors [NS5AI: daclatasvir (DCV) and ledipasvir (LDV)]. The other 3 drugs tested were host-targeting agents (HTAs) including interferon-alpha (IFNα) and cyclophilin inhibitors [Cis: cyclosporin A (CsA) and SCY-635. For multidrug studies, cells were treated with combinations of two or three drugs prior to evaluation of activity. All anti-HCV agents were purchased or kindly provided as described [14].

## Results

**Fig. 1:**
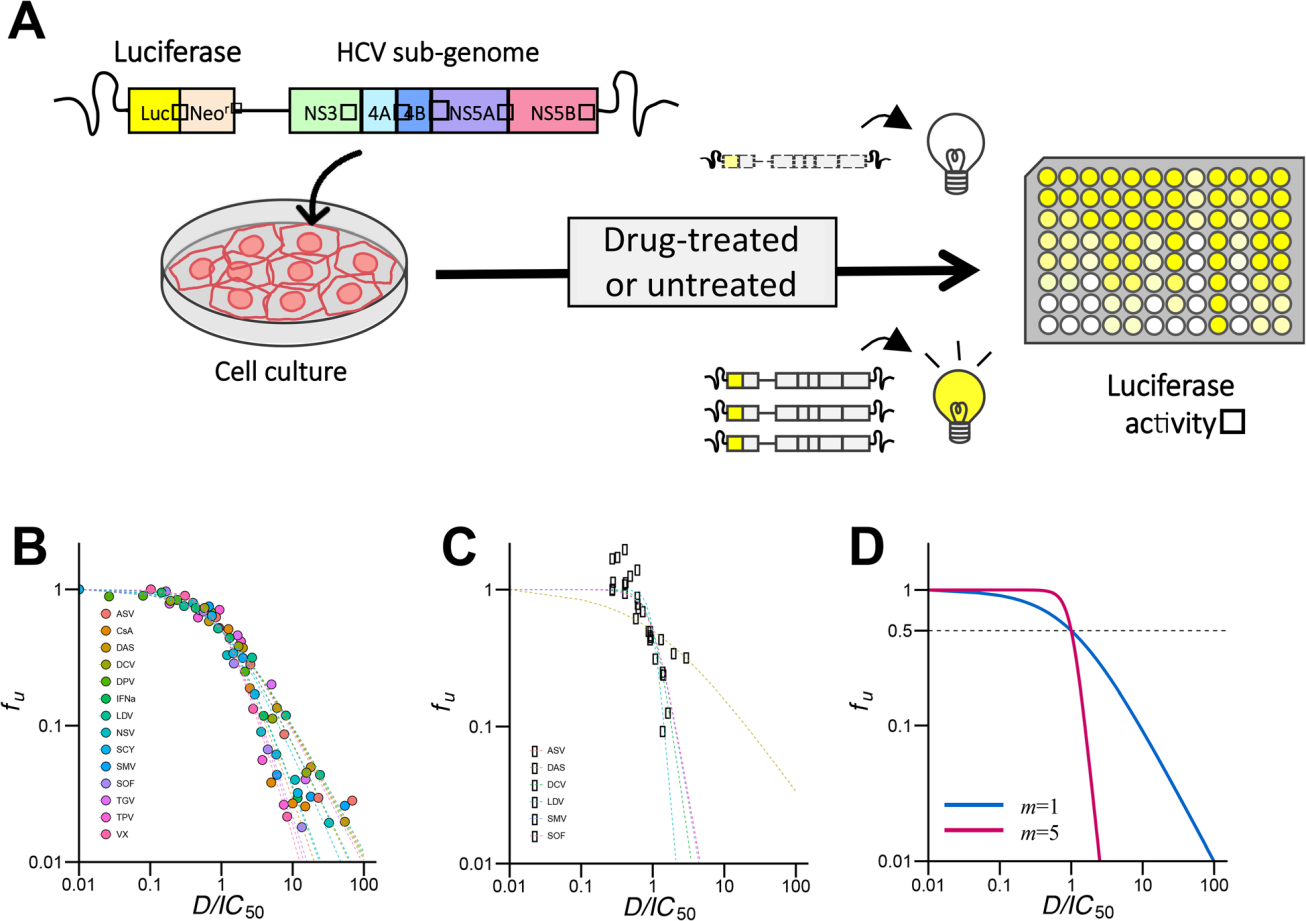
Experimental and mathematical system for quantifying antiviral activity of hepatitis C virus (HCV) drug(s): **a** Schematic representation of the method for quantifying HCV replication level. The assay uses HCV subgenomic replicons for genotype 1b and 2a carrying a fusion of the firefly luciferase gene (Luc) with or without the neomycin phosphotransferase (Neo^r^). The replicons autonomously and persistently replicate in Huh-7.5.1 cells [18, 19]. Cells were incubated for 72 h with or without drug(s) then harvested and luciferase activity detected. Inhibition of HCV replication was quantified as the luciferase activity in drug-treated cells, relative to untreated cells. **b** Log–Log plots of dose-response curves normalized by *IC*_50_ (x-axis), determined from HCV genotype 1b subgenomic replicon assay of NS3 protease inhibitors (PIs; TPV, DPV, ASV, SMV), nucleoside-type NS5B polymerase inhibitor (NI; SOF), non-nucleoside-type NS5B polymerase inhibitors (NNIs; VX, DAS, NSV, TGV), NS5A inhibitors (NS5AIs; DCV, LDV), interferon (IFNα), and cyclophilin inhibitors (CIs; CsA, CSY). Each point represents the mean of three experiments. Least-square regression analysis was used to fit Eq.(1) to the corresponding dose-response curve for estimation of *IC*_50_ and *m* value for each drug against HCV genotype 1b. **c** Log–Log plots of dose-response curves from HCV genotype 2a subgenomic replicon assay of PIs (ASV, SMV), NI (SOF), NNIs (DAS), and NS5AIs (DCV, LDV). Each point represents the mean of three experiments. Least-square regression analysis was used to fit Eq. [1] to the corresponding dose-response curve for each drug against HCV genotype 2a. **d** Dose-response curves for hypothetical drugs with *m* = 1 and 5. Drugs with a higher *m* value show stronger antiviral activity at the same normalized drug concentration

Fig. 1a provides a schematic of the combined experimental and mathematical system that we previously developed for quantifying anti-HCV activity of drug(s) [14]. In the previous study 14 anti-HCV agents were evaluated in mono and combination treatments against HCV genotype 1b [14]. In the current study the same 14 drugs (Table 1) were tested against HCV genotype 1b (Fig. 1b) and HCV genotype 2a (Fig. 1c). Antiviral activity results from mono and combination treatments were used to develop a novel ranking index, the “required concentration index” or RCI (see below). Note that 14 anti-HCV agents include 11 direct-acting antivirals (DAAs) including NS3 protease inhibitors [PIs; telaprevir (TPV), danoprevir (DPV), simeprevir (SMV), and asunaprevir (ASV)], a nucleoside NS5B polymerase inhibitor [NI; sofosbuvir (SOF)], non-nucleoside NS5B polymerase inhibitors [NNIs; VX-222 (VX), dasabuvir (DAS), nesbuvir (NSV), and tegobuvir (TGV)], and NS5A inhibitors [NS5AI; daclatasvir (DCV) and ledipasvir (LDV)] and 3 host-targeting agents (HTAs) included interferon-alpha (IFNα) and cyclophilin inhibitors [CIs; cyclosporin A (CsA) and SCY-635 (SCY)].

**Table 1:** Estimated characteristic parameters of the tested antiviral drugs

Drug	Type	Target	Class	*IC*_50_		*m*		RCI_95_	
				genotype 1b	genotype 2a	genotype 1b	genotype 2a	genotype 1b	genotype 2a
TPV (nM)	DAA	NS3 protease	PI	323.79	–	1.72	–	5.54	–
DPV (nM)	DAA	NS3 protease	PI	1.40	–	0.98	–	20.18	–
SMV (nM)	DAA	NS3 protease	PI	0.45	153.95	1.10	3.10	14.54	2.59
ASV (nM)	DAA	NS3 protease	PI	2.75	665.49	0.97	2.99	20.81	2.68
SOF (nM)	DAA	NS5B polymerase NI	NI	120.48	843.74	1.66	3.02	5.89	2.65
VX (pM)	DAA	NS5B polymerase NNI	NNI	107.58	–	1.81	–	5.08	–
DAS (nM)	DAA	NS5B polymerase NNI	NNI	1.50	7203.98	0.99	0.73	19.57	56.39
NSV (nM)	DAA	NS5B polymerase NNI	NNI	0.25	–	1.19	–	11.87	–
TGV (nM)	DAA	NS5B polymerase NNI	NNI	8.92	–	1.01	–	18.45	–
DCV (nM)	DAA	NS5A	NS5AI	0.10	0.13	1.11	3.68	14.19	2.23
LDV (nM)	DAA	NS5A	NS5AI	0.67	30.80	0.96	6.11	21.48	1.62
IFNα (IU/ml)	HTA	–	IFN	2.56	–	1.43	–	7.84	–
CsA (μg/m)	HTA	Cyclophilin	CI	0.40	–	1.53	–	6.85	–
SCY (μM)	HTA	Cyclophilin	CI	0.34	–	1.45	–	7.62	–

### Ranking anti-HCV mono-drug treatments

As shown in Fig. 1b, c, the antiviral profile of drugs against HCV genotypes 1b and 2a vary widely, suggesting that anti-HCV drugs exhibit strain-dependent effects. The typical dose-response curves of a single antiviral drug can be analyzed using the following hill function [14] (Fig. 1d):

1 $$ {f}_u=\frac{1}{1+{\left(\frac{D}{IC_{50}}\right)}^m}. $$

Here, *f*_*u*_ represents the fraction of infection events unaffected by the drug (i.e., 1 − *f*_*u*_ equals the fraction of drug-affected events). *D* is the drug concentration, *IC*_50_ is the drug concentration that achieves 50% inhibition of activity, and *m* is the slope of the dose-response curve (i.e., Hill coefficient) [14]. Dose-response curves for drugs with higher *m* values show stronger antiviral activity at the same normalized drug concentration so long as the drug concentration is higher than *IC*_50_ (Fig. 1d). Least-square regression analysis was used to fit Eq.(1) to dose-response curves (Fig. 1b, c) and estimate *IC*_50_ and *m* values. Estimated values for each drug against each HCV genotype are summarized in Table 1. The hill function may not accurately fit the dose-response curve at lower drug concentrations (Fig. 1c, especially for doses lower than *IC*_50_). Typical clinical drug concentrations are around 10- to 100-fold of *IC*_50_, therefore it is generally possible to quantify effectiveness of anti-HCV drug(s) with this method especially for such a high drug concentration. As discussed in recent publications [8–14], both *IC*_50_ and *m* values are needed to accurately estimate antiviral drug potency, though only *IC*_50_ is widely used in the drug development field. Since estimated values for each drug differ relative to target HCV genotype, it is important to optimize mono and combination therapy against each genotype.

To characterize efficacy of drugs, we calculated a “required concentration index” (RCI) for each anti-HCV drug against genotype 1b and 2a. Assuming 1 − *f*_*u*_ = *x* inhibition of viral replication, the RCI_*x*_ represents the critical fold increase of *IC*_50_ requiring *x* inhibition of viral replication. Solving Eq.(1) for *D*/*IC*_50_, then RCI_*x*_ is represented as follows:

2 $$ {\mathrm{RCI}}_x=\frac{D_x}{I{C}_{50}}={\left(\frac{1}{f_u}-1\right)}^{\frac{1}{m}}={\left(\frac{x}{1-x}\right)}^{\frac{1}{m}}. $$

**Fig. 2:**
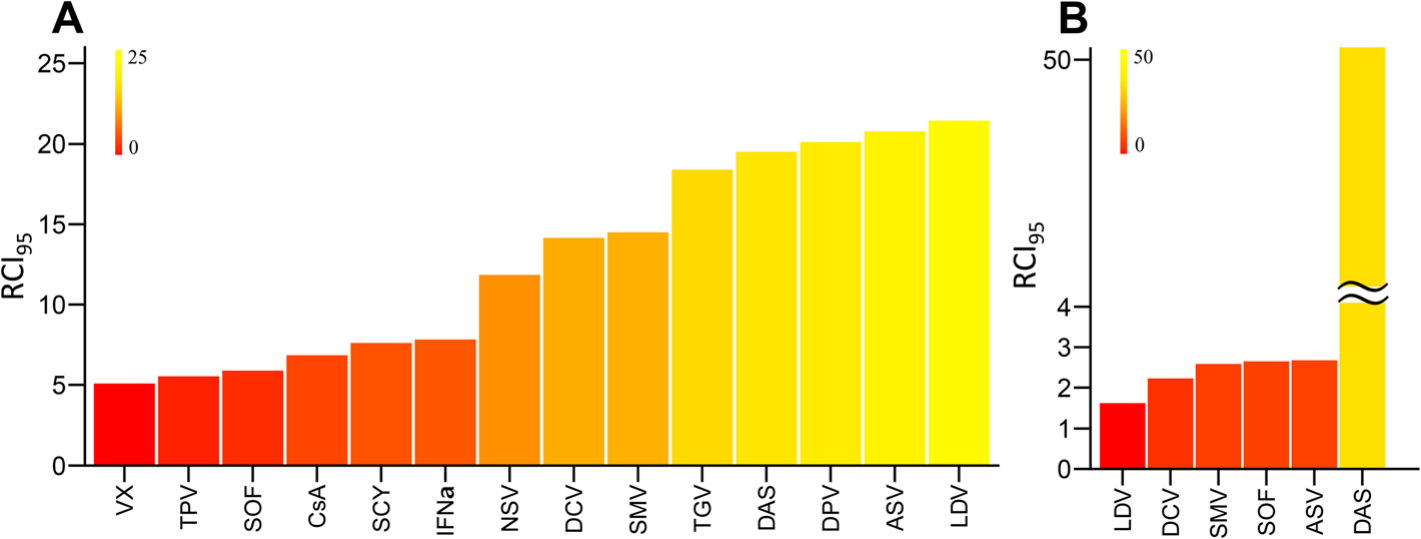
Ranking anti-HCV mono drug treatments against genotypes 1b and 2a: The critical dose of antiviral drug (i.e., fold increase of *IC*_50_) required to inhibit viral replication by 95%, i.e., RCI_95_, was calculated for HCV (**a**) genotype 1b and (**b**) genotype 2a

Here, *D*_*x*_ is the drug concentration required to suppress *x* of viral replication. Drugs with small RCI_x_ values are more efficient inhibitors of HCV replication than drugs with high RCI_x_. Interestingly, high *m* tends to be associated with smaller RCI_x_. By substituting estimated *IC*_50_ and *m* parameters and setting *x* to 0.95 in Eq.(2), we calculated the RCI_x_ required for 95% inhibition of HCV replication (i.e., RCI_95_). We summarize RCI_95_ values of each drug against genotypes 1b and 2a in Fig. 2a, b, respectively. It should be noted that SOF, a nucleoside-type polymerase inhibitor used as a key agent in current and past DAA combinations, was effective in both genotype 1b and 2a, which is consistent with SOF’s known clinical pan-genotypic anti-HCV characteristic [20].

### Ranking anti-HCV multi-drug treatments

**Fig. 3:**
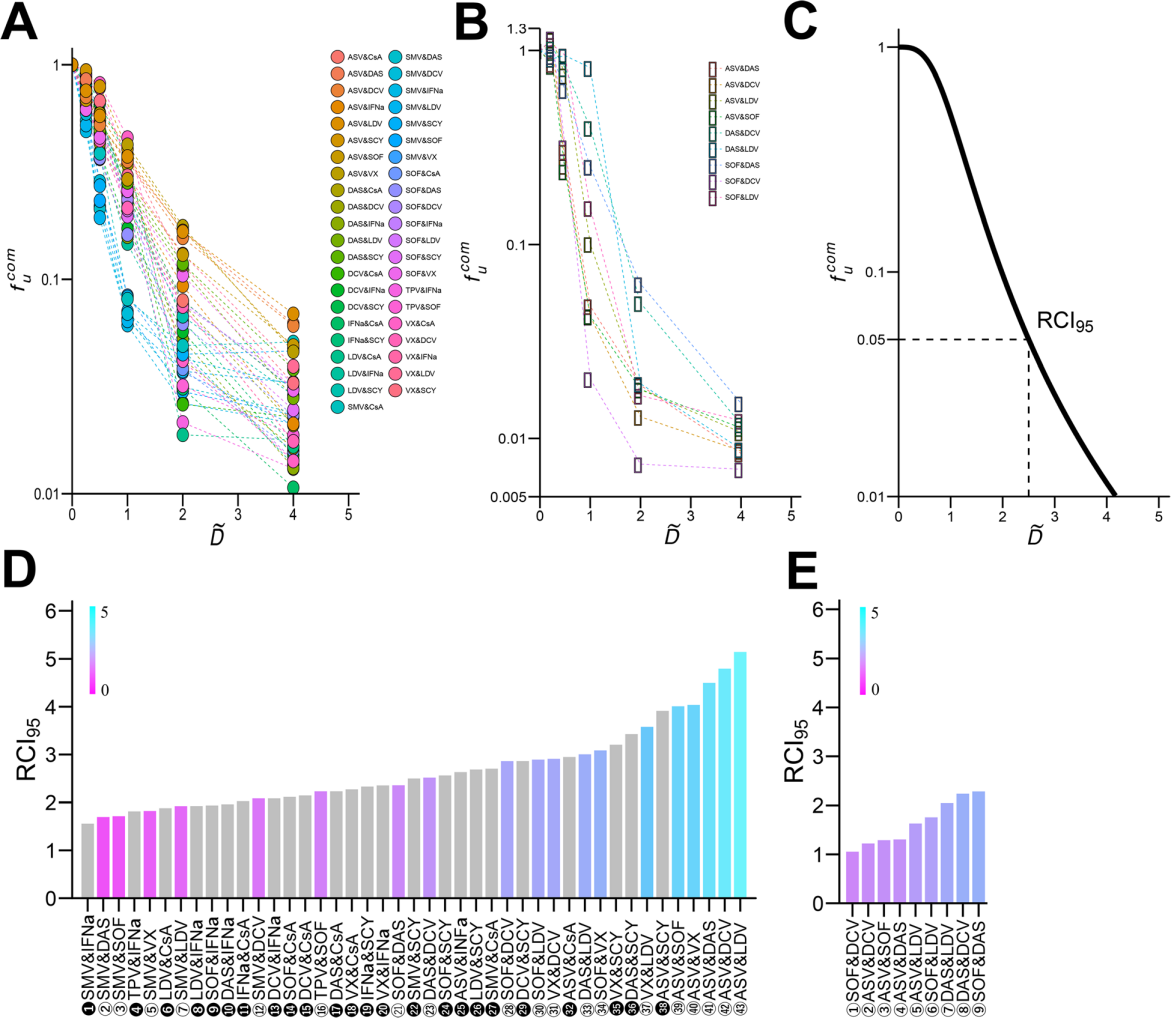
Quantification of anti-HCV double-drug combinations against genotype 1b and 2a: Log–log plots of dose-response curves for 43 (**a**) and 9 (**b**) double-drug combinations of inter-class (or subclass) antiviral drugs against genotype 1b (**a**) and genotype 2a (**b**). Each point represents the mean of four experiments. For comparison of drugs, concentration of each drug was based on a consistent ratio of its *IC*_50_ value. (**c**) Dose-response curve for a hypothetical double-drug combination. RCI_95_ for (double or triple-) drug combinations can be determined from the point where the curve intersects ${f}_u^{\mathrm{com}}=0.05$ (dashed line). (**d**) and (**e**) show RCI_95_ rankings for 43 double-combinations against HCV genotype 1b and 9 double-combinations against HCV genotype 2a, respectively. Combinations with gray bars and black numbers (e.g., ❶) correspond to treatment regimens that include HTAs. Light pink to blue bars and white numbers (e.g., ②) correspond to treatment combinations with DAA-only

**Fig. 4:**
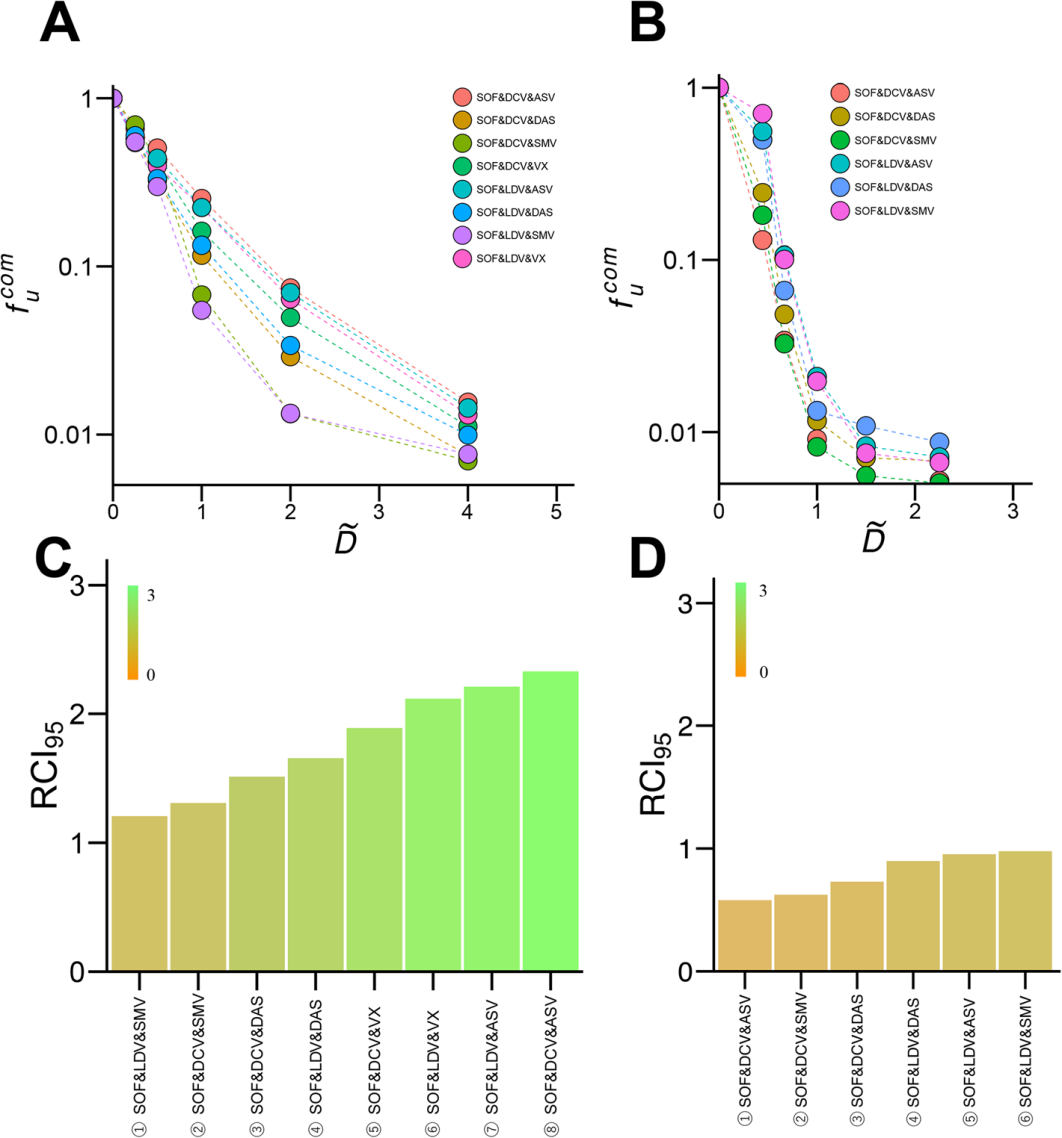
Quantification of anti-HCV triple-drug combinations against genotype 1b and 2a: Log–log plots of dose-response curves for 8 (**a**) and 6 (**b**) triple-drug combinations against HCV genotype 1b (**a**) and 2a (**b**). Each point represents the mean of five experiments for genotype 1b and four experiments for genotype 2a. For comparison of drugs, concentration of each drug was based on a consistent ratio of its *IC*_50_ value. (**c**) and (**d**) show RCI_95_ rankings of the triple-drug combinations against HCV genotype 1b and 2a, respectively

Using the replicon system, the antiviral activity of double- and triple-drug combinations (Fig. 3 & Fig. 4) were investigated using consistent ratios of drug concentrations (i.e., 0.25 × *IC*_50_, 0.5 × *IC*_50_, 1 × *IC*_50_, 2 × *IC*_50_, and 4 × *IC*_50_).

Inhibitory activity was evaluated for 43 double drug combinations against HCV genotype 1b, and, 9 double drug combinations against genotype 2a. Results are shown in Fig. 3a, b, respectively. Here, *D*_*a*_, *D*_*b*_, …, *D*_*i*_ are defined as the concentration of drug a, b, …, i and $I{C}_{50}^a$*,* $I{C}_{50}^b$*, …,* $I{C}_{50}^i$ refer to the corresponding *IC*_50_. Combined drug concentration in these experiments is described as ${D}^{\mathrm{com}}=\left({D}_a,{D}_b,\dots, {D}_i\right)=\left(\overset{\sim }{D}\times I{C}_{50}^a,\overset{\sim }{D}\times I{C}_{50}^b,\dots, \overset{\sim }{D}\times I{C}_{50}^i\right)$, where $\overset{\sim }{D}={D}_a/I{C}_{50}^a={D}_b/I{C}_{50}^b=\dots ={D}_i/I{C}_{50}^i$ is the constant ratio to *IC*_50_ of each combined drug (x-axis of dose-response curves). As shown in Fig. 3c, a similar hill function can be fit to dose-response curves of drug combinations [14]:

3 $$ {f}_u^{\mathrm{com}}=\frac{1}{1+{\left(\frac{\overset{\sim }{D}}{I{C}_{50}^{\mathrm{com}}}\right)}^{m^{\mathrm{com}}}}. $$

Here, ${f}_u^{\mathrm{com}}$ is the fraction of infection events unaffected by the drug combination, $I{C}_{50}^{\mathrm{com}}$ is the constant ratio that inhibits HCV replication by 50%, and *m*^com^ is the Hill coefficient [14]. In Table 2, we summarize estimated parameters, $I{C}_{50}^{\mathrm{com}}$ and *m*^com^, for double-drug combinations.

**Table 2:** Estimated characteristic parameters of the antiviral drug combinations

Drug	${IC}_{50}^{\mathrm{com}}$		*m*^com^		RCI_95_	
	genotype 1b	genotype 2a	genotype 1b	genotype 2a	genotype 1b	genotype 2a
ASV&CsA	0.80	–	2.25	–	2.95	–
ASV&DAS	0.61	0.28	1.48	1.94	4.50	1.30
ASV&DCV	0.59	0.27	1.41	1.97	4.79	1.22
ASV&IFNα	0.53	–	1.84	–	2.63	–
ASV&LDV	0.65	0.47	1.43	2.30	5.14	1.63
ASV&SCY	0.88	–	1.97	–	3.91	–
ASV&SOF	0.54	0.24	1.47	1.75	4.01	1.29
ASV&VX	0.77	–	1.78	–	4.04	–
DAS&CsA	0.60	–	2.23	–	2.23	–
DAS&DCV	0.47	0.78	1.75	2.80	2.52	2.24
DAS&IFNα	0.40	–	1.84	–	1.96	–
DAS&LDV	0.54	0.86	1.72	3.41	3.00	2.05
DAS&SCY	0.57	–	1.64	–	3.43	–
DCV&CsA	0.50	–	2.02	–	2.14	–
DCV&IFNα	0.40	–	1.79	–	2.08	–
DCV&SCY	0.53	–	1.75	–	2.86	–
IFNα&CsA	0.67	–	2.67	–	2.02	–
IFNα&SCY	0.59	–	2.14	–	2.33	–
LDV&CsA	0.43	–	1.99	–	1.88	–
LDV&IFNα	0.38	–	1.82	–	1.93	–
LDV&SCY	0.52	–	1.79	–	2.69	–
SMV&CsA	0.23	–	1.20	–	2.70	–
SMV&DAS	0.18	–	1.31	–	1.70	–
SMV&DCV	0.20	–	1.26	–	2.08	–
SMV&IFNα	0.17	–	1.34	–	1.56	–
SMV&LDV	0.14	–	1.14	–	1.92	–
SMV&SCY	0.19	–	1.14	–	2.50	–
SMV&SOF	0.20	–	1.38	–	1.71	–
SMV&VX	0.24	–	1.45	–	1.82	–
SOF&CsA	0.55	–	2.18	–	2.12	–
SOF&DAS	0.34	0.62	1.53	2.25	2.36	2.28
SOF&DCV	0.47	0.27	1.64	2.14	2.86	1.05
SOF&IFNα	0.37	–	1.77	–	1.94	–
SOF&LDV	0.42	0.52	1.52	2.43	2.89	1.76
SOF&SCY	0.50	–	1.80	–	2.56	–
SOF&VX	0.47	–	1.57	–	3.09	–
TPV&IFNα	0.46	–	2.14	–	1.81	–
TPV&SOF	0.64	–	2.37	–	2.23	–
VX&CsA	0.77	–	2.71	–	2.27	–
VX&DCV	0.47	–	1.61	–	2.91	–
VX&IFNα	0.47	–	1.83	–	2.35	–
VX&LDV	0.59	–	1.63	–	3.58	–
VX&SCY	0.76	–	2.05	–	3.20	–

Similar to mono treatments, the required concentration index for drug combinations is derived as

4 $$ {\mathrm{RCI}}_x={\overset{\sim }{D}}_c=I{C}_{50}^{\mathrm{com}}{\left(\frac{1}{f_u^{\mathrm{com}}}-1\right)}^{\frac{1}{m^{\mathrm{com}}}}=I{C}_{50}^{\mathrm{com}}{\left(\frac{x}{1-x}\right)}^{\frac{1}{m^{\mathrm{com}}}}, $$

The RCI_95_ required for 95% inhibition of HCV replication is extrapolated from the point at which the curve intersects ${f}_u^{\mathrm{com}}=0.05$ (dashed line in Fig. 3c). Note that the critical constant ratio, ${\overset{\sim }{D}}_c$, satisfying Eq.(4) can be uniquely determined. The RCI_95_ values for double-drug combinations against genotype 1b and 2a are summarized in Fig. 3d, e, respectively. RCI_95_ varies depending on drug combination. For genotype 1b, RCI_95_ ranged from 1.56 to 5.14, for genotype 2a RCI_95_ ranged from 1.05 to 2.28. The drug combination with the best anti-HCV profile against genotype 1b is SMV plus IFNα; Fig. 3d❶. This combination used to be the first-in-line anti-HCV drug prior to the development of DAA treatments [17]. Combinations including a non-DAA are presented as gray bars with black number designations. Combinations with DAA-only double treatments are plotted in light pink to blue and designated with white numbers (Fig. 3d). For the DAA-only combinations, one of the most effective treatments against genotype 1b was the combination of SMV and SOF (Fig. 3d③), a primary treatment choice in the early era of DAA-only treatment [16]. A long term first-in-line DAA combination, SOF and LDV (Fig. 3d, e⑥), ranked in the mid-range of efficacy against both genotype 1b and 2a. Most other drug combinations ranked differently against genotype 1b and genotype 2a. ASV plus LDV (Fig. 3d, e⑤) was the least effective DAA-only combination against genotype 1b, but fell in the mid-range for effectiveness against genotype 2a. SOF plus DAS (Fig. 3d, e⑨) ranked in the mid-range against genotype 1b, but ranked lowest against genotype 2a. These trends suggest an overall difference in drug effect depending on the target HCV genotype, and indicate the importance of profiling drugs against each genotype.

Eight triple-DAA treatments were profiled against HCV genotype 1b and 6 triple-combinations were evaluated against genotype 2a (Fig. 4a, b). Triple combination assessments included NS3 protease inhibitor (SMV, ASV) with NS5A inhibitor (DCV, LDV) and NI NS5B polymerase inhibitor (SOF), or NS5A inhibitor with NI NS5B polymerase inhibitor and NNI NS5B polymerase inhibitor (VX, DAS). $I{C}_{50}^{\mathrm{com}}$ and *m*^com^ for triple-drug combinations are summarized in Table 3. We need to note that our experimental assay can detect the range of $0.005<{f}_u^{com}<0.01$ in Fig. 4a, b, whereas it is difficult to measure ${f}_u^{com}<0.005$ in areas of higher drug concentration, reaching to the detection limit of the assay. RCI_95_ values of triple-drug combinations against genotype 1b and 2a are summarized in Fig. 4c, d, respectively. RCI_95_ values ranged from 1.21 to 2.33 for genotype 1b and 0.58 to 0.98 for genotype 2a. Triple combination treatment with SOF, LDV and SMV was most effective against genotype 1b (Fig. 4c①), and least effective against genotype 2a (Fig. 4d⑥). SOF plus DCV and SMV (Fig. 4c②) was also significantly effective against genotype 1b, consistent with the reported clinical efficacy of this triple combination [21, 22]. These results show the optimal combination of drugs to suppress viral replication in vitro, and shed light on the promising drug combinations for improving clinical outcome.

**Table 3:** Estimated characteristic parameters of the antiviral drug combinations

Drug	${IC}_{50}^{\mathrm{com}}$		*m*^com^		RCI_95_	
	genotype 1b	genotype 2a	genotype 1b	genotype 2a	genotype 1b	genotype 2a
SOF&DCV&VX	0.40	–	1.92	–	1.89	–
SOF&DCV&ASV	0.55	0.13	2.03	2.09	2.33	0.58
SOF&DCV&DAS	0.34	0.20	1.97	2.28	1.52	0.73
SOF&DCV&SMV	0.33	0.16	2.14	2.23	1.31	0.62
SOF&LDV&VX	0.45	–	1.90	–	2.12	–
SOF&LDV&ASV	0.46	0.33	1.88	2.88	2.21	0.95
SOF&LDV&DAS	0.35	0.26	1.88	2.49	1.66	0.90
SOF&LDV&SMV	0.26	0.35	1.90	3.04	1.21	0.98

The correlation in ranking between the required concentration index and clinical data suggest that this method could assist with the search for drugs that achieve an efficient antiviral inhibition with different HCV genotypes.

## Discussion

Our study shows that the concentration of drug (calculated as fold of *IC*_50_), that achieves 95% virus inhibition (RCI_95_), highly varied depending on the type of drug and combination with other drugs. RCI_95_ of drugs in mono treatment ranged as much as 4.2 fold in antiviral activity against HCV genotype 1b (Fig. 2a, RCI_95_ = 5.08–21.4). This diversity in RCI_95_ indicates the importance of characterizing more than just the IC_50_ of drugs when predicting antiviral efficacy in clinical settings. In double-drug combinations, RCI_95_ values decreased (Fig. 3d, e) compared with mono treatments (Fig. 2a, b), indicating elevated antiviral activity resulted from combination treatment. The RCI_95_ values of DAA-only double combinations ranged from 1.70 (SMV & DAS) to 5.14 (ASV & LDV) in genotype 1b and from 1.05 (SOF & DCV) to 2.28 (SOF & DAS) in genotype 2a. Thus, the diversity in RCI_95_ is different among genotypes. Genotype differences are probably due differences in replication activity and the varied dependency on target [23, 24].

Triple DAA treatments have become the final strategy for improving treatment outcomes, especially with difficult-to-treat HCV. Triple combinations are also used as a means to shorten treatment periods. Understanding the activity of triple DAA combinations is important in advancing towards worldwide eradication of HCV virus [25–28]. Consistent with ongoing clinical trials which show higher treatment efficacy of triple-drug combinations, triple combinations reduced RCI_95_ beyond double-drug combination levels (Fig. 4c, d). RCI_95_ for triple drug combinations ranged from 1.21 to 2.33 in genotype 1b and from 0.58 to 0.98 in genotype 2a. Interestingly, the RCI_95_ values of selected drugs (SMV, ASV, DCV, LDV, DAS, VX and SOF) were less variable in triple-drug combinations compared with double combinations. RCI_95_ ranged 1.9 fold (1.21 for SOF & LDV & SMV to 2.33 for SOF & DCV & ASV) with triple-drug combinations, 3.0 fold (1.70 for SMV & DAS to 5.14 for ASV & LDV) in double-drug combinations, and 4.2 fold (5.08 for VX to 21.4 for LDV) in single-drug treatment against genotype 1b. These data suggest that multidrug treatments such as triple-drug combinations provide more consistent antiviral effect irrespective of the choice of drugs, yet another advantage of triple combinations.

## Conclusion

In an era of rapidly progressing anti-HCV treatments, selection of the “best” combination treatment is critical to establishing the next generation of anti-HCV treatments against difficult-to-treat HCV and eventually eradicating HCV. We have developed an integrated experimental and mathematical method to evaluate the efficacy of anti-HCV dugs against HCV genotype 1b and 2a. The method was used to score mono- and multi-drug treatment regimens against HCV. This scoring could be used to optimize multidrug treatment regimens prior to clinical entry.

## Data Availability

All data generated or analyzed during this study are included in this published article.

## Abbreviations

HCV: Hepatitis C virus
DAA: Direct acting antiviral
RCI: Required concentration index
TPV: Telaprevir
DPV: Danoprevir
SMV: Simeprevir
ASV: Asunaprevir
SOF: Sofosbuvir
VX: VX-222
DAS: Dasabuvir
NSV: Nesbuvir
TGV: Tegobuvir
DCV: Daclatasvir
LDV: Ledipasvir
IFNα: Interferon-alpha
CsA: Cyclosporin A
SCY: SCY-635
