# Correction to: Required concentration index quantifies effective drug combinations against hepatitis C virus infection

**DOI:** 10.1186/s12976-021-00137-y

**Published:** 2021-01-19

**Authors:** Yusuke Kakizoe, Yoshiki Koizumi, Yukino Ikoma, Hirofumi Ohashi, Takaji Wakita, Shingo Iwami, Koichi Watashi

**Affiliations:** 1 Department of Biology, Faculty of Sciences, Kyushu University, Fukuoka, 812-8581 Japan; 2 Present address: Data Science Group, Advanced Technology Division, INTAGE Inc, Tokyo, 101-8201 Japan; 3 National Center for Global Health and Medicine, Tokyo, 162-8655 Japan; 4 Department of Virology II, National Institute of Infectious Diseases, Tokyo, 162-8640 Japan; 5 Department of Applied Biological Science, Tokyo University of Science, Noda, 278-8510 Japan; 6 Institute for the Advanced Study of Human Biology (ASHBi), Kyoto University, Kyoto, 606-8501 Japan; 7 NEXT-Ganken Program, Japanese Foundation for Cancer Research (JFCR), Tokyo, 135-8550 Japan; 8 Science Groove Inc, Fukuoka, Japan; 9 Institute for Frontier Life and Medical Sciences, Kyoto University, Kyoto, Japan

**Correction to: Theor Biol Med Model (2021) 18:4**

**https://doi.org/10.1186/s12976-020-00135-6**

Following publication of the original article [1], we were notified of a few mismatches between the confirmed figures and tables and the final published versions:

- Additional squares were incorrectly added after the letters in figure 1a
- The points in the graphs of figure 1C and 3B had incorrectly been deleted
- The display of IC_com_50 in the first row of Tables 2 and 3 was not bold

The original paper has been corrected.
